# A viscoelastic–viscoplastic constitutive model for polymer bonded explosives under low impact loading

**DOI:** 10.1038/s41598-022-26525-z

**Published:** 2022-12-17

**Authors:** Youcai Xiao, Zeyu Wang, Ruisheng Wang, Xiaowei Zhang, Chenyang Fan, Zhifang Wei, Yi Sun

**Affiliations:** 1 College of Mechatronic Engineering, North University of China, Taiyuan, 030051 China; 2 State Key Laboratory of Explosion Science and Technology, Beijing Institute of Technology, Beijing, 100081 China; 3 Science and Technology on Electromechanical Dynamic Control Laboratory, Xi’an, 710000 China; 4 Departments of Astronautic Science and Mechanics, Harbin Institute of Technology, Harbin, 150001 China

**Keywords:** Mechanical engineering, Computational science

## Abstract

Viscoplastic work is very important to explosive ignition under impact loading. At present, a large number of constitutive models only consider the viscoelastic and damage behavior of explosives, ignoring the plastic effect under low impact loading. A new viscoelastic–viscoplastic (VE–VP) model was developed and studied to describe the dynamic mechanical behaviors of polymer-bonded explosives (PBXs). The total strain was assumed to be the sum of the viscoelastic (VE) and viscoplastic (VP) components. A generalized Maxwell model was used to determine the VE responses. A VP model was developed by using the classical *J*_2_ rate-dependent model with isotropic hardening. Viscoplastic flow was considered in hyperbolic sinusoidal form. The explicit algorithms of VE model were proposed and assessed by using two different integration methods. The accuracy and efficiency of these two methods are similar at high strain rates. The coupled algorithms of VE–VP model were developed by referring to the classical elasto-viscoplasticity (EVP) provided and using the expression of incremental relaxation modulus. The proposed model was implemented in the ABAQUS using a user-subroutine (VUMAT) to predict the response behaviors of PBX 9501 under low impact loading. Several numerical simulations illustrated the computational efficiency and the accuracy of the proposed methods. The model predictions were compared with experimental data, and reasonable agreement was obtained.

## Introduction

Polymer-bonded explosives (PBXs) comprise energetic crystals that adhere to a polymer binder and can be used in numerous applications, including propellant grains and warheads^1^. Understanding the mechanical behavior and establishing a constitutive model of PBXs are of great interest for defense and commercial applications, and predictive constitutive model descriptions that can be used in finite element simulations of damage and deformation are in demand, with emphasis on understanding the dynamics of the localization phenomena and mechanical failure of PBXs^2^.

The violent reaction induced by low velocity impact (about tens to hundreds meter per second) is not the result of direct shock wave loading, since the pressure generated by the impact is relatively low (20–500 MPa) and the duration time of the pressure is relatively long (50 μs–10 ms). The response of solid explosives under low velocity impact includes several complex processes, such as the localization of plastic work, the conversion from plastic work to heat, the heat conduction, the ignition and the combustion to detonation transition. The split Hopkinson pressure bar (SHPB) test is the simplest and most commonly used method of assessing the sensitivity of low-velocity impacted explosives.

Up to now, many constitutive models have been proposed and applied to study the dynamic mechanical behavior of PBXs^1,3,4^. Dienes et al.^5^ proposed a statistical crack mechanics model (SCRAM) considering anisotropic damage. Base on the SCRAM, Bennett et al.^6^ and Hackett et al.^7^ developed a viscoelastic cracking constitutive model (Visco-SCRAM) to simulate the combined VE and brittle damage behavior of PBX 9501. Liu et al.^8^ developed a VE–VP damage model to describe the stress–strain responses and damage behaviors of PBX based on Bodner–Partom viscoplasticity. Liu et al.^9^ developed the constitutive model which incorporates a porosity model and a VE–VP damage model to study the strain rate and pressure-related deformation and damage of PBXs. Le et al.^10^ proposed a constitutive model by using a VE model with damage and pressure-dependent plasticity to describe the static mechanical behavior of PBXs based on isotropic hardening law. Clements et al.^11^ developed a constitutive theory to describe the dynamic mechanical behavior of PBX 9501 based on Mori and Tanaka’s effective medium theory. Yang et al.^12^ developed a damaged viscoelastic–plastic (VE–P) model to predict dynamic mechanical behavior based on both micro-crack and micro-void damage evolution laws.

Buechler et al.^13^ developed a new implementation of a constitutive model incorporate Visco-SCRAM and linear VE with isotropic damage evolution to represent PBXs in finite element simulations of thermomechanical response under quasi-static compression. Tong et al.^14^ developed a nonlinear VE model to describe the quasi-static behavior of PBX using two stress-dependent nonlinear functions within the hereditary integral formulation of viscoelasticity. Jung et al.^15^ proposed a nonlinear VE constitutive model of solid propellants under quasi-static loading. In the model, the damage was accounted for by the modulus decrease due to dewetting. Swanson et al.^16^ developed a constitutive formulation for rubbery PBXs by using linear viscoelasticity as well as large-deformation elastic–plastic (E–P) model under low strain rates. Zubelewicz et al.^17^ developed an elastic–viscoplastic (E–VP) model with the rates of the homogeneous VE deformation and the growth of micro-damages.

PBX 9501 is a particulate composite nominally consisting of 95 wt% HMX (octrahydro-1,3,5,7-tetranitro-1,3,5,7-tetrazocine) crystals embedded in a polymer binder consisting of 50% ESTANE and 50% BDNPA/F). PBX 9501 is not perfectly VE, because stressing the samples can cause permanent damage through plastic grain debonding (or dewetting), crystal fracture, or viscoplastic (VP) work. PBXs exhibit complex inelastic behaviors that are time and rate dependent at all stages under impact loading. However, the study on constitutive model hasn’s considered both viscoelasticity and viscoplasticity under impact loading. To simulate these dynamic mechanical behaviors requires a constitutive model that combines nonlinear VE effects with viscoplasticity. Although there has been a great deal of research in both viscoelasticity and viscoplasticity, attempts to develop constitutive relations that combine these two areas have been limited.

This paper details a new VE–VP constitutive model is implemented and studied to represent the PBX 9501 in finite element simulations of dynamic mechanic response. A computational algorithm for the coupled VE–VP model is presented. The computational algorithm is detailed and studied. We predict the dynamic responses of PBX 9501 and perform a comparison with the experimental results.

## Constitutive model

### Kinematics

The Kinematic of this material model are based on small strain theory. The Cauchy stress and total strain can be decomposed into the hydrostatic and deviatoric components as follows^13^:

1 $$\left\{ \begin{gathered} \varepsilon_{ij} = e_{ij} + \varepsilon_{V} \delta_{ij} \hfill \\ \sigma_{ij} = S_{ij} + \sigma_{V} \delta_{ij} \hfill \\ \end{gathered} \right.,$$

where $e_{ij}$ is the deviatoric strain tensor, and $S_{ij}$ is the deviatoric stress tensor, and $\varepsilon_{V}$ is the hydrostatic strain tensor, and $\sigma_{V}$ is the hydrostatic stress tensor, and $\delta_{ij}$ is the Kronecker delta, $\delta_{ij} = 1$ for *i* = *j* and 0 otherwise.

The total strain is decomposed into two parts, a VE strain $\varepsilon_{ij}^{ve}$ and a VP strain $\varepsilon_{ij}^{vp}$, which is expressed as follows^18^:

2 $$\varepsilon_{ij} = \varepsilon_{ij}^{ve} + \varepsilon_{ij}^{vp} ,$$

where $\varepsilon_{ij}^{ve}$ is the VE component of the strain and $\varepsilon_{ij}^{vp}$ is the portion of the VP strain.

### Viscoelasticity

The VE response is expressed in a hereditary integral form, which is Boltzmann’s form if the VE model is linear and usually the Schapery form for nonlinear VE models^19–21^. Based on Boltzmann’s superposition principle^22^, the integral form of the VE stress is as follows:

3 $$\sigma_{ij} \left( t \right) = \int_{0}^{t} {L_{ijkl} \left( {t - \tau } \right)\frac{{\partial \varepsilon_{ij}^{ve} \left( \tau \right)}}{\partial \left( \tau \right)}} d\tau .$$

For an isotropic material, the fourth-rank relaxation tensor is written as follows:

4 $$L_{ijkl}^{{}} \left( t \right) = G(t)\left( {\delta_{ij} \delta_{kl} + \delta_{il} \delta_{jk} } \right) + \left( {K(t) - \frac{2}{3}G(t)} \right)\delta_{ij} \delta_{kl} ,$$

where *G*(*t*) and *K*(*t*) represent the rate dependent shear and bulk relaxation functions, respectively.

A Prony series representation was used to model the shear and bulk relaxation function. *G*(*t*) and *K*(*t*) can be expressed as a Prony series of shear elements and bulk elements:

5 $$\left\{ \begin{gathered} G\left( t \right) = G^{\left( \infty \right)} + \sum\limits_{n = 1}^{N} {G^{\left( n \right)} \exp \left( { - \frac{t}{{\tau^{\left( n \right)} }}} \right)} \hfill \\ K\left( t \right) = K^{\left( \infty \right)} + \sum\limits_{n = 1}^{N} {K^{\left( n \right)} \exp \left( { - \frac{t}{{\tau^{\left( n \right)} }}} \right)} \hfill \\ \end{gathered} \right.,$$

where *G*^(*n*)^ and *K*^(*n*)^ represent the shear and bulk stiffness, respectively, $\tau^{\left( n \right)}$ is the shear and bulk relaxation times, *G*^(∞)^ and *K*^(∞)^ are the long-term elastic shear and bulk moduli, respectively, and *N* is the number of Maxwell elements.

By substituting Eqs. (4) and (5) into Eq. (3), the Cauchy stress $\sigma_{ij}$ can be rewritten as follows:

6 $$\sigma_{ij} \left( t \right) = \underbrace {{\int_{0}^{t} {2G\left( {t - \tau } \right)} \frac{{\partial e_{ij}^{ve} \left( \tau \right)}}{\partial \tau }d\tau }}_{{S_{ij} \left( t \right) = {\text{dev}}\sigma_{ij} \left( t \right)}} + \underbrace {{\int_{0}^{t} {3K\left( {t - \tau } \right)} \frac{{\partial \varepsilon_{V}^{ve} \left( \tau \right)}}{\partial \tau }d\tau }}_{{\sigma_{V} \left( t \right) = \frac{1}{3}{\text{tr}}\sigma_{ij} \left( t \right)}}\delta_{ij} .$$

The deviatoric ($S_{ij} \left( t \right)$) and hydrostatic ($\sigma_{V} \left( t \right)$) parts of the stress tensor ($\sigma_{ij} \left( t \right)$) may be expressed as the sum of the elastic and the Maxwell components:

7 $$\left\{ \begin{gathered} S_{ij} \left( t \right) = S_{ij}^{el} \left( t \right) + \sum\limits_{n = 1}^{N} {S_{ij}^{(n)} } \hfill \\ \sigma_{V} \left( t \right) = \sigma_{V}^{el} \left( t \right) + \sum\limits_{n = 1}^{N} {\sigma_{V}^{(m)} } \hfill \\ \end{gathered} \right..$$

The elastic element is given as follows:

8 $$\left\{ \begin{gathered} S_{ij}^{el} (t) = 2G_{\infty } e_{ij}^{ve} (t) \hfill \\ \sigma_{V}^{el} (t) = 3K_{\infty } \varepsilon_{V}^{ve} (t) \hfill \\ \end{gathered} \right..$$

Each Maxwell element is given as follows:

9 $$\left\{ \begin{array}{*{20}l} S_{ij}^{\left( n \right)} = 2G^{\left( n \right)} \int_{0}^{t} {\exp \left( { - \frac{t - \tau }{{\tau^{\left( n \right)} }}} \right)} \frac{{\partial e_{ij}^{ve} (\tau )}}{\partial \tau }d\tau \hfill \\ \sigma_{V}^{\left( n \right)} (t) = 3K^{\left( n \right)} \int_{0}^{t} {\exp \left( { - \frac{t - \tau }{{\tau^{\left( n \right)} }}} \right)} \frac{{\partial \varepsilon_{V}^{ve} (\tau )}}{\partial \tau }d\tau \hfill \\ \end{array} \right..$$

### Viscoplasticity

The viscous stress ($\sigma_{v}$) considered in the subsequent formula derivation is a power-law function, which is defined as follows^23^:

10 $$\sigma_{v} = k\dot{p}^{m} ,$$

where *k* and *m* are material constants. The constant *m* is called the material strain rate sensitivity. The stress is given as follows^24^:

11 $$\sigma = \sigma_{y} + r(p) + \sigma_{v} = \sigma_{y} + r(p) + k\dot{p}^{m} ,$$

where $\sigma_{y}$ is the initial yield stress, $r\left( p \right)$ is the hardening stress, and *p* is the effective plastic strain. In the VP model, the uniaxial stress depends on the yield stress, the hardening of the yield stress, and the plastic strain rate.

According to the Eq. (11), the plastic strain rate is

12 $$\dot{p} = \left( {\frac{{\sigma - r(p) - \sigma_{y} }}{k}} \right)^{1/m} .$$

For a von Mises material, the uniaxial stress is identical to the effective stress and similar for the effective plastic strain rate. Equation (12) can therefore be written as follows:

13 $$\dot{p} = \left( {\frac{{\sqrt {\tfrac{2}{3}S_{ij} :S_{ij} } - r(p) - \sigma_{y} }}{k}} \right)^{{{1 \mathord{\left/ {\vphantom {1 m}} \right. \kern-0pt} m}}} .$$

According of the normality hypothesis of plasticity, we also write the normality hypothesis for viscoplasticity as follows:

14 $${\dot{\mathbf{\varepsilon }}}^{vp} = \lambda \frac{\partial f}{{\partial {{\varvec{\upsigma}}}}}.$$

For the isotropic hardening, the von Mises yield function is given as follows^25^:

15 $$f\left( {{{\varvec{\upsigma}}},R} \right) = \sigma_{eq} - \sigma_{y} - R(p)\;{\text{and}}\;\sigma_{eq} = \sqrt {\frac{3}{2}{\mathbf{S}}:{\mathbf{S}}} ,$$

where $\sigma_{eq}$ is the von Mises equivalent stress.

Equation (14) can be rewritten as

16 $${\dot{\mathbf{\varepsilon }}}^{vp} = \dot{\lambda }\frac{\partial f}{{\partial {{\varvec{\upsigma}}}}} = \dot{\lambda }\frac{3}{2}\frac{{\mathbf{S}}}{{\sqrt {\tfrac{3}{2}{\mathbf{S}}:{\mathbf{S}}} }}.$$

The effective plastic strain is an internal variable that keeps track of the past history of the material and is linked to the VP law as follows:

17 $$p(t) = \int_{0}^{t} {\dot{p}(\tau )} d\tau .$$

The effective plastic strain is defined as

18 $$\dot{p} = \left( {\frac{2}{3}{{\varvec{\upvarepsilon}}}^{vp} :{{\varvec{\upvarepsilon}}}^{vp} } \right)^{{{1 \mathord{\left/ {\vphantom {1 2}} \right. \kern-0pt} 2}}} .$$

The effective plastic strain rate is obtained by

19 $$\dot{p} = \frac{2}{3}\left( {\dot{\lambda }\frac{3}{2}\frac{{\mathbf{S}}}{{\sqrt {\tfrac{3}{2}{\mathbf{S}}:{\mathbf{S}}} }}:\dot{\lambda }\frac{3}{2}\frac{{\mathbf{S}}}{{\sqrt {\tfrac{3}{2}{\mathbf{S}}:{\mathbf{S}}} }}} \right)^{{{1 \mathord{\left/ {\vphantom {1 2}} \right. \kern-0pt} 2}}} = \dot{\lambda }.$$

By substituting Eq. (19) into Eq. (16), the VP strain rate can be obtained as

20 $${\dot{\mathbf{\varepsilon }}}^{vp} = \frac{3}{2}\left( {\frac{{\sqrt {\tfrac{3}{2}{\mathbf{S}}:{\mathbf{S}}} - r(p) - \sigma_{y} }}{k}} \right)^{{{1 \mathord{\left/ {\vphantom {1 m}} \right. \kern-0pt} m}}} \frac{{\mathbf{S}}}{{\sqrt {\tfrac{3}{2}{\mathbf{S}}:{\mathbf{S}}} }}.$$

The VP multiplier $\dot{p}$ is determined by the following conditions^23^:

21 $$\dot{p} = \phi \left( {\Delta p,r} \right) = a\sinh \left[ {\beta \left( {\sqrt {\tfrac{3}{2}{\mathbf{S}}:{\mathbf{S}}} - r(p) - \sigma_{y} } \right)} \right],{\text{ if}}\;f > \, 0,$$

22 $$\dot{p} = 0,{\text{ if}}\;f < \, 0,$$

where $a$ and $\beta$ represent the VP modulus and exponent, respectively.

The hardening function *r*(*p*) is a linear law which is defined as^26^:

23 $$r\left( p \right) = hp,$$

where *h* is the hardening modulus.

## Computational algorithm

### VE model computational algorithm

The numerical algorithm proposed in this section is suitable for VE behavior. For a generic time interval $\left[ {t_{u} ,t_{u + 1} } \right]$, with the values of the variables at $t_{u}$ known and the total strain increment $\Delta {{\varvec{\upvarepsilon}}}$ given, the aim of the algorithm detailed hereafter is to compute the values of the variables at time $t_{u + 1}$. Given ∆*t* and the strain increment $\Delta \varepsilon_{ij}^{ve}$, $S_{ij}^{\left( n \right)} \left( {t_{u + 1} } \right)$ and $\sigma_{V}^{\left( n \right)} (t_{u + 1} )$ at time $t_{u + 1}$in the VE model can be obtained as follows^27^:

24 $$\left\{ \begin{gathered} S_{ij}^{\left( n \right)} \left( {t_{u + 1} } \right) = \exp \left( { - \frac{\Delta t}{{\tau^{\left( n \right)} }}} \right)S_{ij}^{\left( n \right)} + 2G^{{_{\left( n \right)} }} \int_{{t_{u} }}^{{t_{u + 1} }} {\exp \left( { - \frac{{t - t_{u + 1} }}{{\tau^{\left( n \right)} }}} \right)} \frac{{\partial e_{ij}^{ve} (\tau )}}{\partial \tau }d\tau \hfill \\ \sigma_{V}^{\left( n \right)} (t_{u + 1} ) = \exp \left( { - \frac{\Delta t}{{\tau^{\left( n \right)} }}} \right)\sigma_{V}^{\left( n \right)} + 3K^{\left( n \right)} \int_{{t_{u} }}^{{t_{u + 1} }} {\exp \left( { - \frac{{t - t_{u + 1} }}{{\tau^{\left( n \right)} }}} \right)} \frac{{\partial \varepsilon_{V} (\tau )}}{\partial \tau }d\tau \hfill \\ \end{gathered} \right..$$

Two approaches are considered to compute the integrals over $\left[ {t_{u} ,t_{u + 1} } \right]$.

If $\Delta \varepsilon_{ij} = \dot{\varepsilon }_{ij} \left( t \right)\Delta t$, integration of Eq. (24) leads to the following:

25 $$\left\{ \begin{gathered} S_{ij}^{\left( n \right)} \left( {t_{u + 1} } \right) = \exp \left( { - \frac{\Delta t}{{\tau^{\left( n \right)} }}} \right)S_{ij}^{\left( n \right)} + 2G^{{_{\left( n \right)} }} \left[ {1 - \exp \left( { - \frac{\Delta t}{{\tau^{\left( n \right)} }}} \right)} \right]\frac{{\tau^{\left( n \right)} }}{\Delta t}\Delta e_{ij}^{ve} \hfill \\ \sigma_{V}^{\left( n \right)} (t_{u + 1} ) = \exp \left( { - \frac{\Delta t}{{\tau^{\left( n \right)} }}} \right)\sigma_{V}^{\left( n \right)} + 3K^{\left( n \right)} \left[ {1 - \exp \left( { - \frac{\Delta t}{{\tau^{\left( n \right)} }}} \right)} \right]\frac{{\tau^{\left( n \right)} }}{\Delta t}\Delta \varepsilon_{V} \hfill \\ \end{gathered} \right..$$

Based on the mid-rectangle rule^28^, the numerical computation of the integrals in Eq. (24) leads to the following:

26 $$\left\{ \begin{gathered} S_{ij}^{\left( n \right)} \left( {t_{u + 1} } \right) = \exp \left( { - \frac{\Delta t}{{\tau^{\left( n \right)} }}} \right)S_{ij}^{\left( n \right)} + 2G^{{_{\left( n \right)} }} \exp \left( { - \frac{\Delta t}{{2\tau^{\left( n \right)} }}} \right)\Delta e_{ij}^{ve} \hfill \\ \sigma_{V}^{\left( n \right)} (t_{u + 1} ) = \exp \left( { - \frac{\Delta t}{{\tau^{\left( n \right)} }}} \right)\sigma_{V}^{\left( n \right)} + 3K^{\left( n \right)} \exp \left( { - \frac{\Delta t}{{2\tau^{\left( n \right)} }}} \right)\Delta \varepsilon_{V} \hfill \\ \end{gathered} \right..$$

Equations (25) and (26) can be rearranged as follows:

27 $$\left\{ \begin{gathered} S_{ij}^{\left( n \right)} \left( {t_{u + 1} } \right) = \exp \left( { - \frac{\Delta t}{{\tau^{\left( n \right)} }}} \right)S_{ij}^{\left( n \right)} + 2\tilde{G}^{\left( n \right)} \Delta e_{ij}^{ve} \hfill \\ \sigma_{V}^{\left( n \right)} (t_{u + 1} ) = \exp \left( { - \frac{\Delta t}{{\tau^{\left( n \right)} }}} \right)\sigma_{V}^{\left( n \right)} + 3\tilde{K}^{\left( n \right)} \Delta \varepsilon_{V} \hfill \\ \end{gathered} \right.,$$

where

28 $$\left\{ \begin{gathered} \tilde{G}^{{_{\left( n \right)} }} = \left[ {1 - \exp \left( { - \frac{\Delta t}{{\tau^{\left( n \right)} }}} \right)} \right]\frac{{g^{\left( n \right)} }}{\Delta t} \hfill \\ \tilde{K}^{\left( n \right)} = \left[ {1 - \exp \left( { - \frac{\Delta t}{{\tau^{\left( n \right)} }}} \right)} \right]\frac{{k^{\left( n \right)} }}{\Delta t} \hfill \\ \end{gathered} \right.\;{\text{or}}\;\left\{ \begin{gathered} \tilde{G}^{{_{\left( n \right)} }} = \exp \left( { - \frac{\Delta t}{{2\tau^{\left( n \right)} }}} \right) \hfill \\ \tilde{K}^{\left( n \right)} = \exp \left( { - \frac{\Delta t}{{2\tau^{\left( n \right)} }}} \right) \hfill \\ \end{gathered} \right..$$

Equation (7) can be written in the following incremental form:

29 $$\left\{ \begin{gathered} S_{ij}^{{}} \left( {t_{u + 1} } \right) = S_{ij}^{el} \left( {t_{u} } \right) + 2G^{\left( \infty \right)} \Delta e_{ij}^{ve} + \sum\limits_{n = 1}^{N} {\exp \left( {\frac{ - \Delta t}{{\tau^{\left( n \right)} }}} \right)S_{ij}^{(n)} \left( {t_{u} } \right)} + \sum\limits_{n = 1}^{N} {2G^{\left( n \right)} \tilde{G}^{\left( n \right)} \Delta e_{ij}^{ve} } \hfill \\ \sigma_{V} \left( {t_{u + 1} } \right) = \sigma_{V}^{el} \left( {t_{u} } \right) + 3K^{\left( \infty \right)} \Delta \varepsilon_{V}^{ve} + \sum\limits_{n = 1}^{N} {\exp \left( {\frac{ - \Delta t}{{\tau^{\left( n \right)} }}} \right)S_{ij}^{(n)} \left( {t_{u} } \right)} + \sum\limits_{n = 1}^{N} {3K^{\left( n \right)} \tilde{K}^{\left( n \right)} \Delta \varepsilon_{V}^{ve} } \hfill \\ \end{gathered} \right..$$

The incremental relaxation moduli $\tilde{G}$ and $\tilde{K}$ are defined as follows^26,29^:

30 $$\left\{ \begin{gathered} \tilde{G}\left( {\Delta t} \right) = G^{(\infty )} + \sum\limits_{n = 1}^{N} {G^{\left( n \right)} \tilde{G}^{{^{\left( n \right)} }} \left( {\Delta t} \right)} \hfill \\ \tilde{K}\left( {\Delta t} \right) = K^{(\infty )} + \sum\limits_{n = 1}^{N} {K^{\left( n \right)} \tilde{K}^{{^{\left( n \right)} }} \left( {\Delta t} \right)} \hfill \\ \end{gathered} \right..$$

The influence of the choice between the two definitions of $\tilde{G}$ and $\tilde{K}$ is assessed in the following work.

### Coupled VE–VP model computational algorithm

It is based on a combination of techniques used separately for VE and elastic–viscoplastic (EVP) models. If the trial stress does not exceed the yield stress, the new stress is set equal to the trial stress. The trial stress can be written as follows:

31 $$\begin{aligned} S_{ij}^{trial} \left( {t_{u + 1} } \right) = & S_{ij}^{\left( \infty \right)} \left( {t_{u} } \right) + 2G^{\left( \infty \right)} \Delta e_{ij}^{ve} \\ & + \sum\limits_{n = 1}^{N} {\exp \left( {\frac{ - \Delta t}{{\tau^{\left( n \right)} }}} \right)S_{ij}^{\left( n \right)} \left( {t_{u} } \right)} \\ & + \sum\limits_{n = 1}^{N} {2G^{\left( n \right)} \frac{{\tau^{\left( n \right)} }}{\Delta t}\left[ {1 - \exp \left( {\frac{ - \Delta t}{{\tau^{\left( n \right)} }}} \right)} \right]\Delta e_{ij}^{ve} } , \\ \end{aligned}$$

32 $$\begin{aligned} \sigma_{V}^{trial} \left( {t_{u + 1} } \right) = & \sigma_{V}^{\left( \infty \right)} \left( {t_{u} } \right) + 3K^{\left( \infty \right)} \Delta \varepsilon_{V}^{el} \\ & + \sum\limits_{n = 1}^{N} {\exp \left( {\frac{ - \Delta t}{{\tau^{\left( n \right)} }}} \right)\sigma_{V}^{\left( n \right)} \left( {t_{u} } \right)} \\ & + \sum\limits_{n = 1}^{N} {3K^{\left( n \right)} \frac{{\tau^{\left( n \right)} }}{\Delta t}\left[ {1 - \exp \left( {\frac{ - \Delta t}{{\tau^{\left( n \right)} }}} \right)} \right]\Delta \varepsilon_{V}^{ve} } . \\ \end{aligned}$$

The new stress at *t*_*u*+1_ can be given by:

33 $$\sigma_{ij} (t_{u + 1} ) = \sigma_{ij}^{trial} (t_{u + 1} ).$$

If the yield stress is exceeded, viscoplasticity occurs in the increment. We then write the incremental analogs of the rate equations as^29^

34 $$\sigma_{ij}^{{}} (t_{u + 1} ) = \sigma_{ij}^{trial} (t_{u} ) - \tilde{L}_{ijkl} :\Delta \varepsilon_{kl}^{vp} ,$$

where $\tilde{L}_{ijkl}^{{}} \left( t \right) = \tilde{G}(t)\left( {\delta_{ij} \delta_{kl} + \delta_{il} \delta_{jk} } \right) + \left( {\tilde{K}(t) - \frac{2}{3}\tilde{G}(t)} \right)\delta_{ij} \delta_{kl}$ represents the incremental relaxation moduli. The relation between Cauchy and trial stresses remains form-identical to EVP, except that the predictor is VE and the relaxation modulus is a function of the time increment.

In the VP model, the effective plastic strain rate is

35 $$\dot{p} = \phi \left( {\sigma_{eq} ,\Delta p} \right) = a\sinh \left[ {\beta \left( {\sqrt {\tfrac{3}{2}{\mathbf{S}}:{\mathbf{S}}} - r(p) - \sigma_{y} } \right)} \right].$$

The effective plastic strain increments are computed according to the backward Euler method as follows:

36 $$\Delta p = \phi \left( {\sigma ,r} \right)\Delta t.$$

This implies that

37 $$\psi \left( {\Delta p,r} \right) = \Delta p - \phi \left( {\sigma ,r} \right)\Delta t = 0.$$

Here, the VP strain tensor is written as follows:

38 $$\Delta \varepsilon_{ij}^{vp} = \Delta pn_{ij} .$$

Equation (34) can be written as follows:

39 $$\sigma_{ij}^{new} (t_{u + 1} ) = \sigma_{ij}^{trial} (t_{u + 1} ) - 2\tilde{G}\Delta \overline{\varepsilon }_{vp} n_{ij} .$$

Using the integrated flow rule, together with the Mises definition of the flow direction $n_{ij}$, this becomes

40 $$\left( {\sigma_{eq} \left( {t^{n + 1} } \right) + 3\tilde{G}\Delta \overline{\varepsilon }_{vp} } \right)n_{ij} \left( {t^{n + 1} } \right) = \sigma_{eq}^{trial} \left( {t^{n + 1} } \right)n_{ij}^{trial} \left( {t^{n + 1} } \right).$$

This implies that

41 $$\sigma_{eq}^{trial} \left( {t^{n + 1} } \right) - 3\tilde{G}\Delta p - \sigma_{eq} \left( {t^{n + 1} } \right) = 0.$$

By Eqs. (37) and (41) if *f* is positive, time integration of the VE–VP law simply implies that an iterative solution of two scalar equations can be obtained:

42 $$\left\{ \begin{gathered} \sigma_{eq}^{trial} \left( {t^{n + 1} } \right) - 3\tilde{G}\Delta p - \sigma_{eq} \left( {t^{n + 1} } \right) = 0 \hfill \\ \Delta p - \phi \left( {\Delta p,r} \right)\Delta t = 0 \hfill \\ \end{gathered} \right..$$

The two unknows, $\sigma_{eq} \left( {t^{n + 1} } \right)$ and $\Delta p$, can be obtained by solving these equations.

We solve these by the Newton–Raphson method. We have the following two scalar equations:

43 $$\left\{ \begin{array}{*{20}l} \psi_{\alpha } \left( {\sigma_{eq} ,\Delta p} \right) = \sigma_{eq}^{trial} \left( {t^{n + 1} } \right) - 3\tilde{G}\Delta p - \sigma_{eq} \left( {t^{n + 1} } \right) = 0 \hfill \\ \psi_{\gamma } \left( {\sigma_{eq} ,\Delta p} \right) = \Delta p - \phi \left( {\sigma_{eq} ,\Delta p} \right)\Delta t = 0 \hfill \\ \end{array} \right..$$

At each iteration (*r*), the equations can be written as follows:

44 $$\left\{ \begin{array}{*{20}l} \psi_{\alpha }^{r} + \left( {\frac{{\partial \psi_{\alpha } }}{\partial \Delta p}} \right)^{r} d\Delta p + \left( {\frac{{\partial \psi_{\alpha } }}{{\partial \sigma_{eq} }}} \right)^{r} d\sigma_{eq} = 0 \hfill \\ \psi_{\gamma }^{r} + \left( { - \frac{{\partial \psi_{\gamma } }}{\partial \Delta p}} \right)^{r} d\Delta p + \left( { - \frac{{\partial \psi_{\gamma } }}{{\partial \sigma_{eq} }}} \right)^{r} d\sigma_{eq} = 0 \hfill \\ \end{array} \right..$$

We obtain:

45 $$\left\{ \begin{array}{*{20}l} \psi_{\alpha }^{{}} + \frac{{\partial \psi_{\alpha } }}{\partial \Delta p}d\Delta p + \frac{{\partial \psi_{\alpha } }}{{\partial \sigma_{eq} }}d\sigma_{eq} = 0 \hfill \\ \psi_{\gamma }^{{}} - 3\tilde{G}d\Delta p - d\sigma_{eq} = 0 \hfill \\ \end{array} \right.,$$

where $$\frac{{\partial \psi_{\alpha } }}{\partial \Delta p} = 1 - \frac{\partial \phi }{{\partial \Delta p}}\Delta t = 1 - \phi_{\Delta p} \Delta t$$, and $$\frac{{\partial \psi_{\alpha } }}{{\partial \sigma_{eq} }} = - \frac{{\partial \phi_{{}} }}{{\partial \sigma_{eq} }}\Delta t = - \phi_{\sigma } \Delta t$$.

Substituting Eq. (45) the second into the first gives the following:

46 $$\psi_{\gamma } + \left( {1 - \phi_{\Delta p} \Delta t} \right)d\Delta p - \phi_{\sigma } \Delta t\left( {\psi_{\alpha } - 3\tilde{G}d\Delta p} \right) = 0.$$

We obtain:

47 $$d\Delta p = \frac{{\phi_{\sigma } \psi_{\alpha } \Delta t - \psi_{\gamma } }}{{1 - \phi_{\Delta p} \Delta t + 3\tilde{G}\phi_{\sigma } \Delta t}}.$$

At iteration (*r* + 1) step, the $\Delta p$ is obtained as

48 $$\Delta p^{{\left( {r + 1} \right)}} = \Delta p^{\left( r \right)} + d\Delta p.$$

### Computation framework

**Figure 1:**
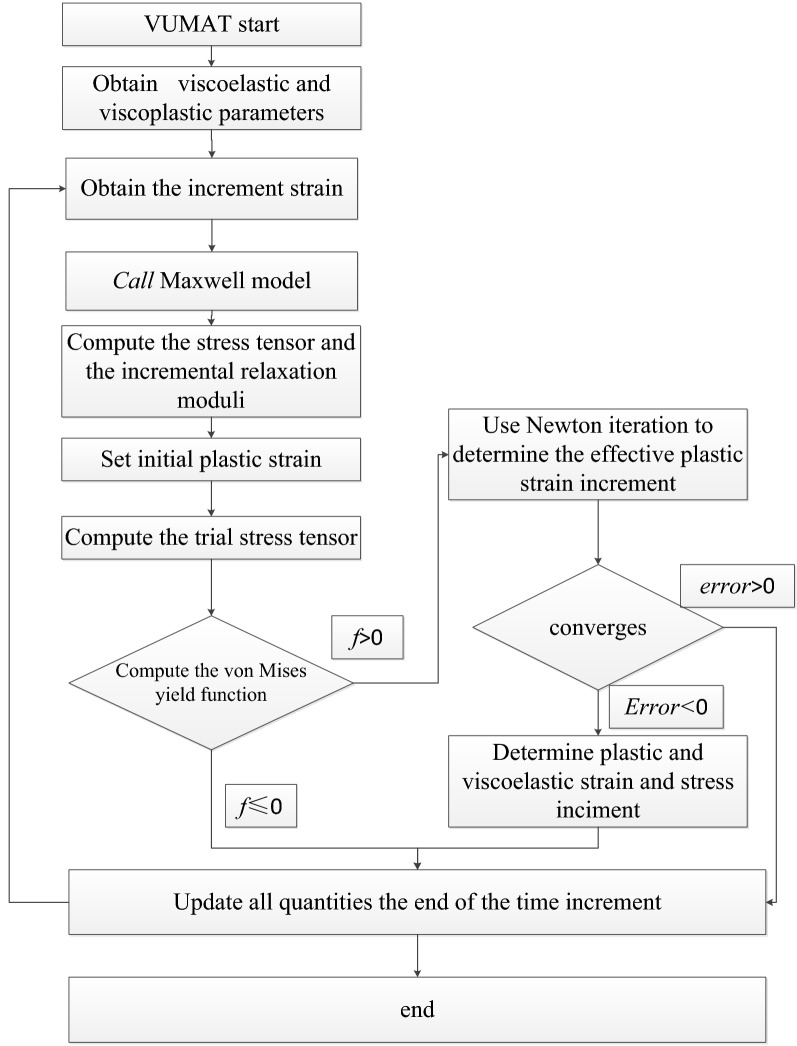
Framework of the VE–VP model.

The numerical algorithm was implemented in the finite element program ABAQUS by coding the user material subroutine VUMAT. The flow chart is shown in Fig. 1.

At the beginning of each time step, the strain increment ($\Delta \varepsilon_{ij} = \Delta \varepsilon_{ij}^{ve}$, $\Delta \varepsilon_{ij}^{vp} = 0$) was imported. The trial stresses were calculated by using Eqs. (31) and (32). Then the yield function was calculated by Eq. (15). If *f* < 0, the stress state was within the yield stress space and yield criterion is not met; then the trial results were real stresses in this time step. Otherwise (*f* > 0), the yield criterion was met, and the trial stresses were calculated through VE–VP algorithm in Table 1.

**Table 1:** VE–VP algorithm.

**Input:** $\Delta e_{ij}^{ve}$, $\Delta \varepsilon_{V}^{ve}$, $\Delta t$; **Params:** $G^{\left( n \right)}$, $K^{\left( n \right)}$, $g^{\left( n \right)}$, $k^{\left( n \right)}$, $\sigma_{y}$, *h*, $\beta$, and *a* **Output:** ${{\varvec{\upvarepsilon}}}^{vp}$, ${{\varvec{\upvarepsilon}}}^{ve}$, $S_{ij}^{{}} \left( {t_{u + 1} } \right)$, $\sigma_{V}^{{}} \left( {t_{u + 1} } \right)$
1: Read the strain tensor at the (*u* + 1)*th* step 2: Compute the trial stress tensor using Eqs. (25) and (26) 3: Compute the incremental relaxation modulus: $$\left\{ \begin{array}{*{20}l} \tilde{G}^{{_{\left( n \right)} }} = \left[ {1 - \exp \left( { - \frac{\Delta t}{{\tau^{\left( n \right)} }}} \right)} \right]\frac{{g^{\left( n \right)} }}{\Delta t} \hfill \\ \tilde{K}^{\left( n \right)} = \left[ {1 - \exp \left( { - \frac{\Delta t}{{\tau^{\left( n \right)} }}} \right)} \right]\frac{{k^{\left( n \right)} }}{\Delta t} \hfill \\ \tilde{G} = G^{(\infty )} + \sum\limits_{n = 1}^{N} {G^{\left( n \right)} \tilde{G}^{{^{\left( n \right)} }} } \hfill \\ \tilde{K} = K^{(\infty )} + \sum\limits_{n = 1}^{N} {K^{\left( n \right)} \tilde{K}^{{^{\left( n \right)} }} } \hfill \\ \end{array} \right.$$ 3: Compute the von Mises yield function *f*: $f = \sigma_{eq}^{trial} - \sigma_{y} = \left( {\frac{3}{2}S_{ij}^{trial} :S_{ij}^{trial} } \right) - \sigma_{y}$ 4: If $f \ge 0$, compute the plastic strain rate; otherwise, go to step (1) 5: Use New iteration to compute the effective plastic strain increment as follows: $$\left\{ \begin{array}{*{20}l} \psi_{\alpha } = \sigma_{eq}^{trial} - 3\tilde{G}\Delta p - \sigma_{eq} \hfill \\ \psi_{\gamma } = \Delta p - \phi \Delta t \hfill \\ \phi_{\Delta p} = - 3\tilde{G}a\beta \cosh \left[ {\beta \left( {\sigma_{eq}^{trial} - 3\tilde{G}\Delta p - hp - \sigma_{y} } \right)} \right] \hfill \\ \phi_{\sigma } = - a\beta \cosh \left[ {\beta \left( {\sigma_{eq}^{trial} - 3\tilde{G}\Delta p - hp - \sigma_{y} } \right)} \right] \hfill \\ d\Delta p = \frac{{\phi_{eq} \psi_{\sigma } \Delta t - \psi_{\varepsilon } }}{{1 - \phi_{\Delta p} \Delta t + 3\tilde{G}\phi_{eq} \Delta t}} \hfill \\ \Delta p^{{\left( {r + 1} \right)}} = \Delta p^{(r)} + d\Delta p \hfill \\ \end{array} \right.$$ 6: Compute the nominal VP strain tensor as follows: $$\left\{ \begin{array}{*{20}l} {{\varvec{\upvarepsilon}}}^{vp} = \frac{3}{2}\Delta p\frac{{{\mathbf{S}}^{trial} }}{{\sigma_{eq}^{trial} }} \hfill \\ {{\varvec{\upvarepsilon}}}^{ve\_new} = {{\varvec{\upvarepsilon}}} - {{\varvec{\upvarepsilon}}}^{vp} \hfill \\ \end{array} \right.$$ 7: Compute the nominal stress tensor: $$\begin{aligned} S_{{ij}}^{{new}} \left( {t_{{u + 1}} } \right) = & S_{{ij}}^{{\left( \infty \right)}} \left( {t_{u} } \right) + 2G^{{\left( \infty \right)}} \Delta e_{{ij}}^{{ve\_new}} + \sum\limits_{{n = 1}}^{N} {\exp \left( {\frac{{ - \Delta t}}{{\tau ^{{\left( n \right)}} }}} \right)S_{{ij}}^{{\left( n \right)}} \left( {t_{u} } \right)} \\ & + \sum\limits_{{n = 1}}^{N} {2g^{{\left( n \right)}} \frac{{g^{{\left( n \right)}} }}{{\Delta t}}\left[ {1 - \exp \left( {\frac{{ - \Delta t}}{{\tau ^{{\left( n \right)}} }}} \right)} \right]\Delta e_{{ij}}^{{ve\_new}} } \\ \end{aligned}$$ $$\begin{aligned} \sigma _{V}^{{new}} \left( {t_{{u + 1}} } \right) = & \sigma _{V}^{{\left( \infty \right)}} \left( {t_{u} } \right) + 3K^{{\left( \infty \right)}} \Delta \varepsilon _{V}^{{ve\_new}} + \sum\limits_{{n = 1}}^{N} {\exp \left( {\frac{{ - \Delta t}}{{\tau ^{{\left( n \right)}} }}} \right)\sigma _{V}^{{\left( n \right)}} \left( {t_{u} } \right)} \\ & + \sum\limits_{{n = 1}}^{N} {3K^{{\left( n \right)}} \frac{{\tau ^{{\left( n \right)}} }}{{\Delta t}}\left[ {1 - \exp \left( {\frac{{ - \Delta t}}{{\tau ^{{\left( n \right)}} }}} \right)} \right]\Delta \varepsilon _{V}^{{ve\_new}} } \\ \end{aligned}$$

## Model validation

### Material parameters for PBX 9501

**Figure 2:**
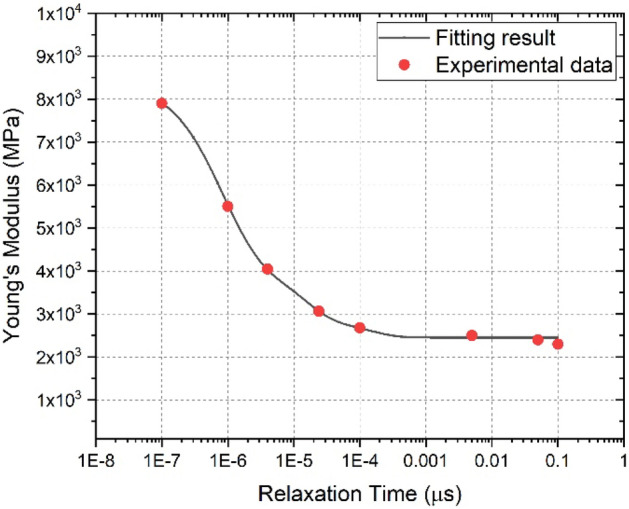
Young’s modulus versus relaxation time data for PBX 9501.

In the VE–VP model, the five Maxwell components have been used to describe the dynamic VE behavior. VE material parameters are obtained in two ways. By means of the time-temperature superposition principle, the master curve of *E*(*t*) versus *t* was constructed using relaxation tests at different temperatures. The material parameters of PBX 9501 are obtained by fitting the modulus master curve. Figure 2 shows the master Young’s modulus curve of PBX 9501. The relaxation times for a GMM were obtained from the relationship that the relaxation times were approximately one-tenth the reciprocal strain rate of the test values. Figure 2 shows the experimental data for the relaxation time and the fit to the data for PBX 9501. The material parameters for PBX 9501 associated with the VE model are given in Table 2.

**Table 2:** Young’s modulus and relaxation parameter for PBX 9501.

Element	1	2	3	4	5
*E*^(n)^ (MPa)	451.88	1355.12	2362.1	1787.5	2454.4
*τ*^(n)^ (s)	1.37 × 10^−4^	1.37 × 10^−5^	1.37 × 10^−6^	5 × 10^−7^	∞

An inverse method is proposed for parameter identification in VP deformation. The inverse solution procedure consists of an optimization method allowing the adjustment of the parameters so that the calculated response matched that measured in a uniaxial compressive test.

We consider a monotonic uniaxial compression test such that $\sigma_{11}$ is the only non-zero component. The von Mises equivalent stress is defined as follows:

49 $$\sigma_{eq} = \sqrt {\frac{3}{2}{\mathbf{S}}:{\mathbf{S}}} = \sigma_{11} .$$

For the hyperbolic plastic flow, the VP function given by Eq. (50) is as follows:

50 $$\dot{p} = \phi \left( {\Delta p,r} \right) = a\sinh \left[ {\beta \left( {\sigma_{eq} - r - \sigma_{y} } \right)} \right].$$

**Figure 3:**
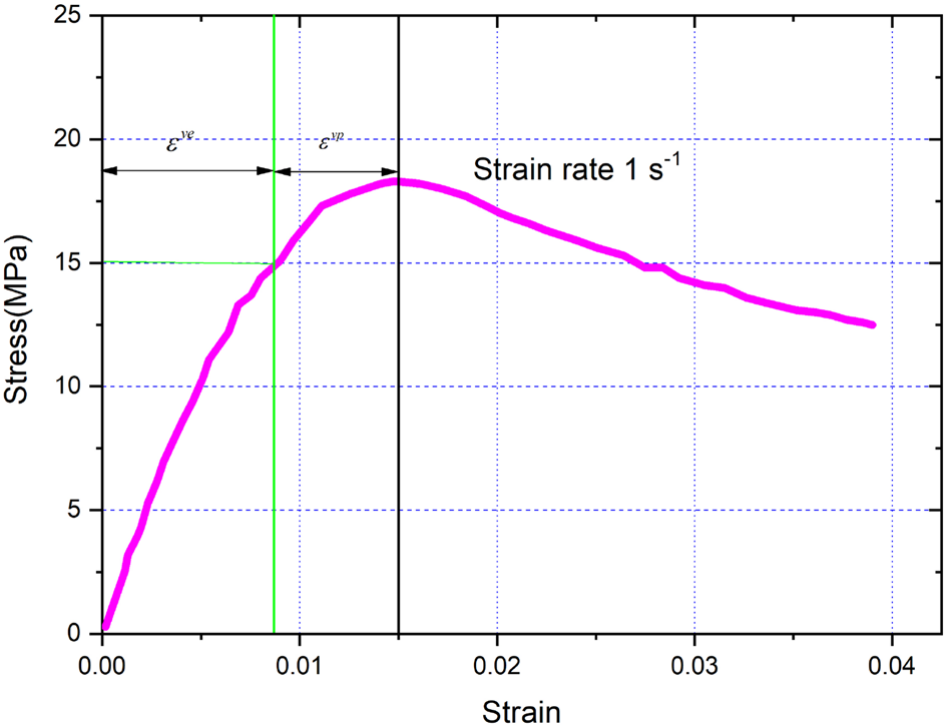
The stress–strain curves for PBX 9501 under the quasi-static compressive tests.

Finally, using kinematic hardening, the effective stress is written as follows:

51 $$\left\{ \begin{array}{*{20}l} \sigma_{eq}^{cal} = arc\left( {\sinh \left( {\frac{{\dot{p}}}{a}} \right)} \right)/\beta + hp + \sigma_{y} \hfill \\ p = \varepsilon - \frac{{\sigma^{\exp } }}{{\tilde{E}}} \hfill \\ \dot{p} = \frac{{p_{u + 1} - p_{u} }}{\Delta t} = \dot{\varepsilon }\frac{{p_{u + 1} - p_{u} }}{{\varepsilon_{u + 1} - \varepsilon_{u} }} \hfill \\ \tilde{E}\left( {\Delta t} \right) = E^{(\infty )} + \sum\limits_{n = 1}^{N} {E^{\left( n \right)} \exp \left( { - \frac{\Delta t}{{2\tau^{\left( n \right)} }}} \right)} \hfill \\ \end{array} \right.,$$

where $\sigma^{\exp }$ is the experimentally measured stress under the quasi-static compressive tests (Fig. 3).

In the VP formulation, four parameters need to be obtained: $\sigma_{y}$, *h*, *a* and *β*. The initial yield stress $\sigma_{y}$ is considered to be rate independent, and $\sigma_{y}$ was obtained under strain rate of 1/s. The optimization minimizes an objective function that is formed from the square of the difference between the measured and simulated stress responses for the considered uniaxial compressive test. Figure 3 shows the stress–strain curve under strain rate of 1/s. The material parameters for PBX 9501 associated with the VP model is given in Table 3.

**Table 3:** VP model parameter for PBX 9501.

*ν*	$\sigma_{y}$ (MPa)	*h* (MPa)	*a*	$\beta$
0.3	15	19	2060	0.0357

### Verification for linear viscoelasticity

**Figure 4:**
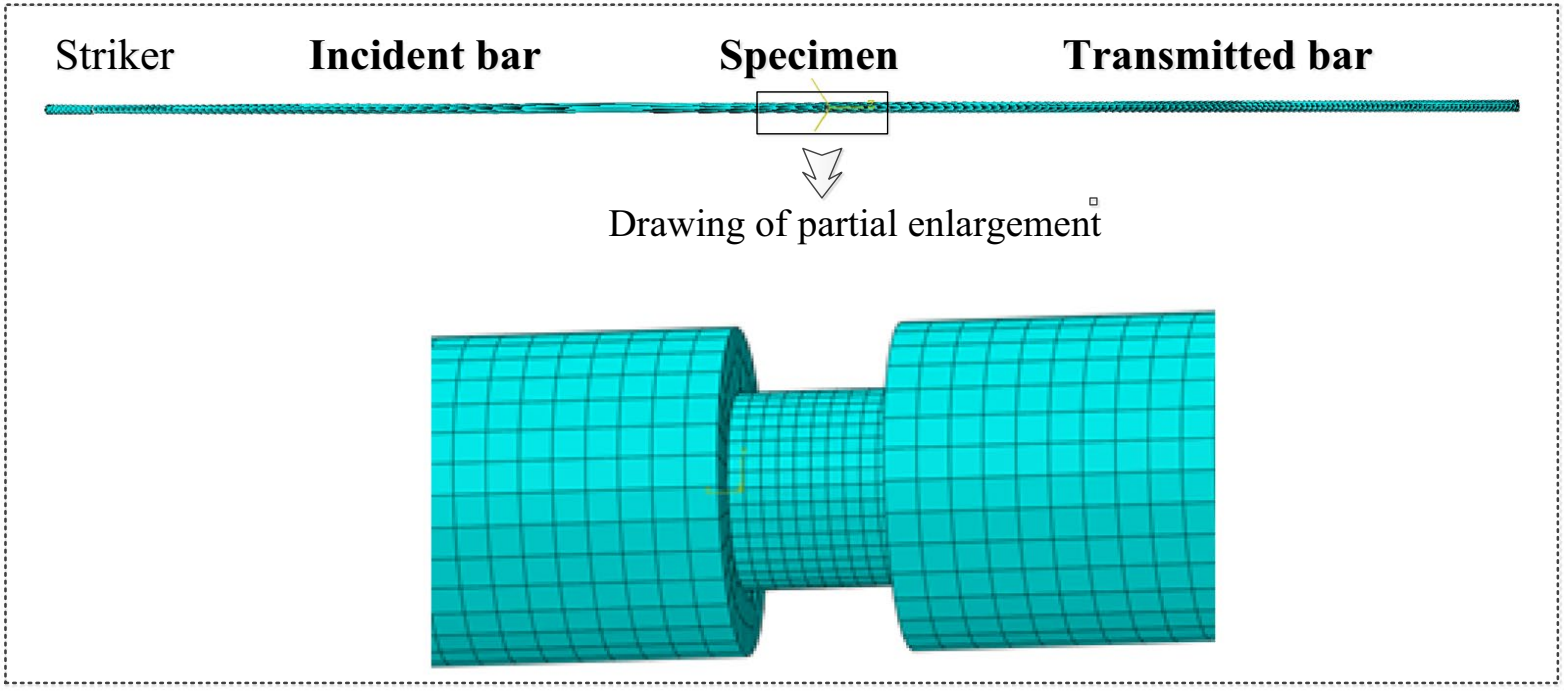
Finite element meshes.

The split Hopkinson pressure bar (SHPB) technique is widely used to determine the dynamic properties of PBX^30^. To verify the simulation code and the effectiveness of the material constitutive law, we first performed simulations with the developed material laws and the existing linear VE model in ABAQUS to obtain the dynamic compressive responses of the PBX 9501. The SHPB system consists of the striker, an incident bar, a transmission bar and an absorption bar. All bars were made of aluminum, of which the Young’s modulus and density were 73 GPa and 2.7 × 10^3^ kg/m^3^, respectively, and the diameters of all the bars were 9.18 mm. The length of the impact bar was 75 mm, and the length of the incident and transmission bars were 1000 mm. The diameter of the specimen was 6.35 mm, and the thickness was 3.15 mm. The SHPB system is partitioned by hexahedron elements, and the mesh size of the specimen is 0.7 mm. The mesh size of the all bar is 1.2 mm, as shown in Fig. 4. Contact constraints are imposed to prevent interpenetration of the system components. The material parameters were obtained from Table 2, where the incremental time was 8.85 × 10^−8^ s.

**Figure 5:**
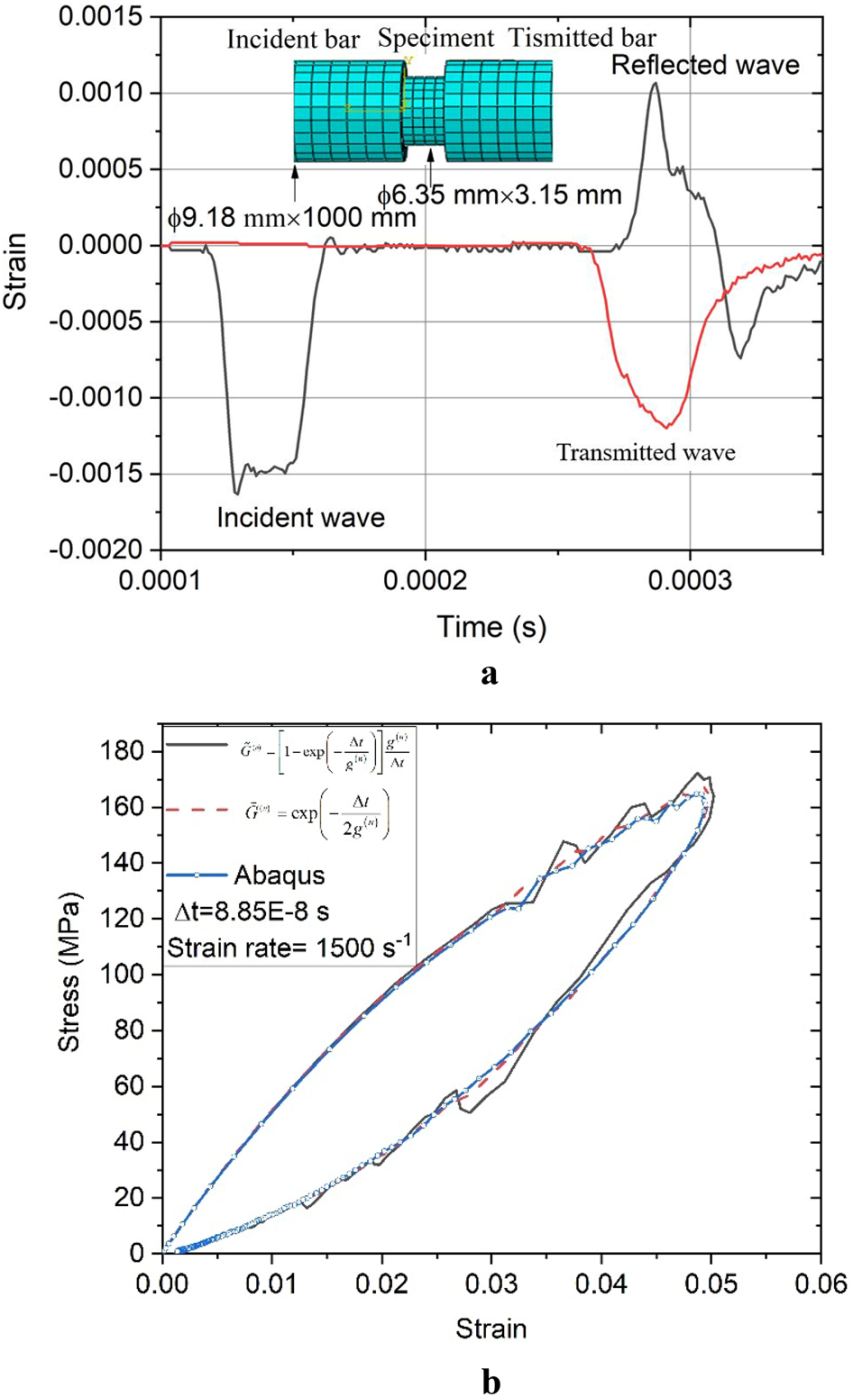
(**a**) Incident, reflected, and transmitted wave from the dynamic compressive simulation of the PBX 9501 using the VE model. (**b**) Comparison of the numerical results calculated by the proposed model and the linear VE material model in ABAQUS.

Incident, reflected, and transmitted waves in the bars are shown in Fig. 5a. We compared the stress–strain curves in the compression direction calculated by the developed material models and the ABAQUS model. The stress–strain curves of the three methods are shown in Fig. 5b. The results obtained with the proposed VE constitutive law agreed fairly well with the results calculated using the linear VE material law in ABAQUS.

**Figure 6:**
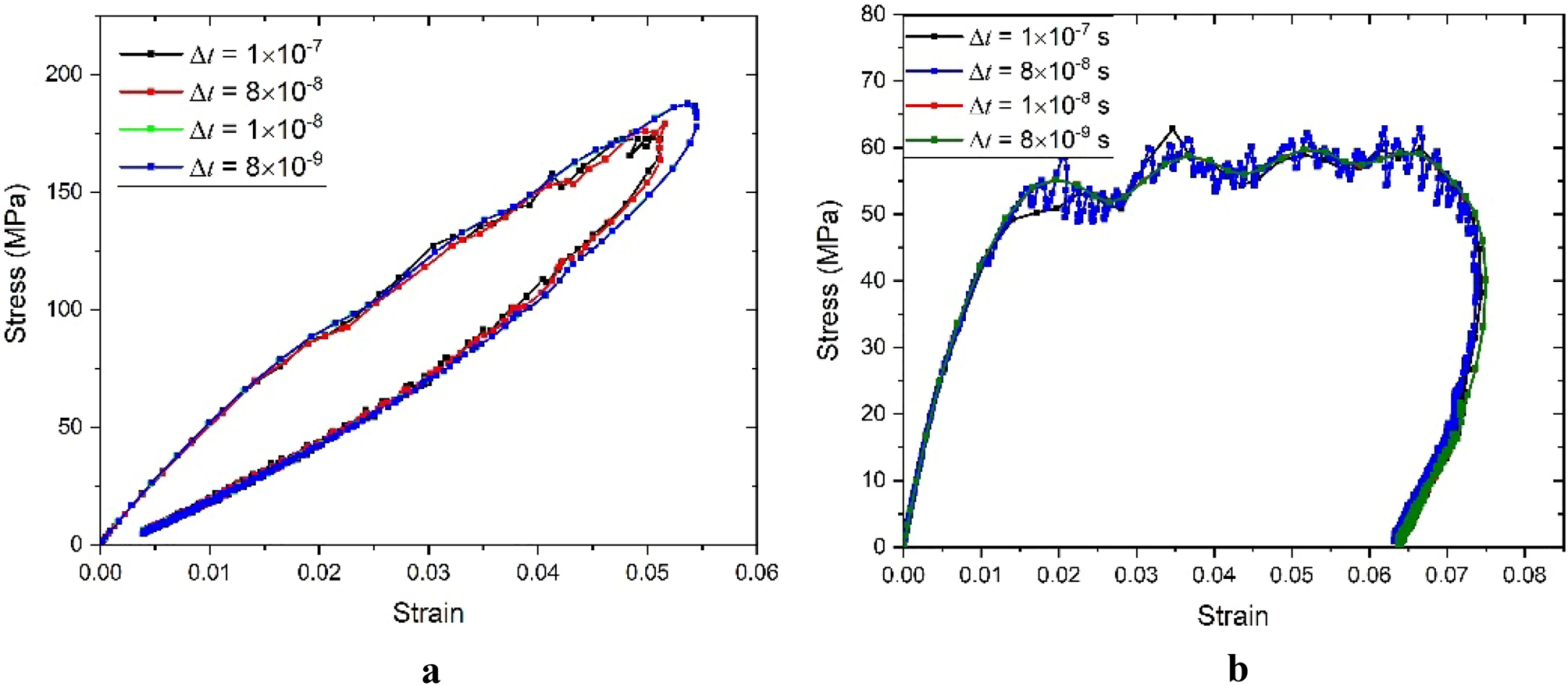
Numerical simulation of SHPB test under different Δ*t*: (**a**) VE model; (**b**) VE–VP model.

Figure 6 shows a comparison of the numerical time integration schemes. The time of the incident wave is 50 µs. By decreasing the time increment, we note that the two definitions of the incremental relaxation modulus lead to very similar and rather accurate results. Due to small Δt in higher strain rate, both integration methods can be used to predict the dynamic mechanical behavior of materials. An important consequence is that if the total strain rate is constant and VP strains do not evolve, then the Cauchy stress computed with the first integration method is insensitive to the value of Δt.

### Comparison of the VE/EVP/VE–VP models

**Figure 7:**
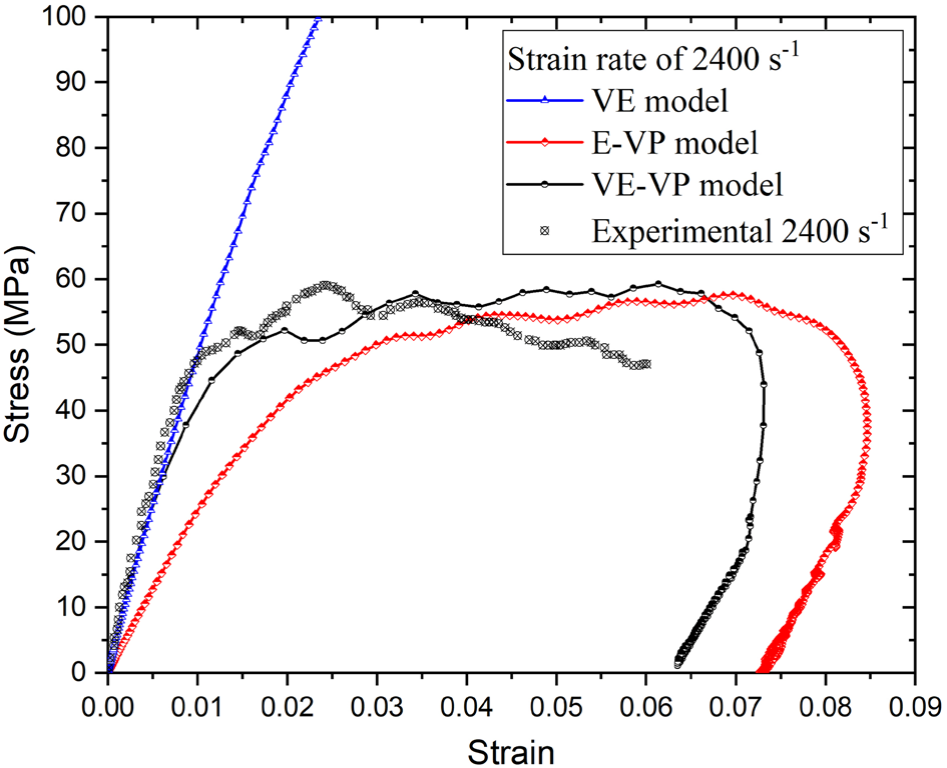
Comparison of the VE, EVP, and VE–VP models.

The VE/EVP/VE-VP model was the characterization of the dynamic mechanical properties of the PBX 9501. The material parameters are shown in Tables 2 and 3. As shown in Fig. 7, the stress–strain curves of the PBX 9501 at a strain rate of 2200/s were calculated for the VE, EVP, and VE–VP models. The comparison of the experimental results and the simulation results in Fig. 7 shows that the VP effect caused the VE and plastic stages to be smoothly connected. Compared with the simulation results of the three models, VE–VP can better describe the dynamic mechanical properties of PBX 9501. The VE–VP and EVP responses were very similar in the VP stage. As expected, the initial slopes of the VE–VP and VE curves coincided in the VE stage.

### Uniaxial compression at various strain rates

**Figure 8:**
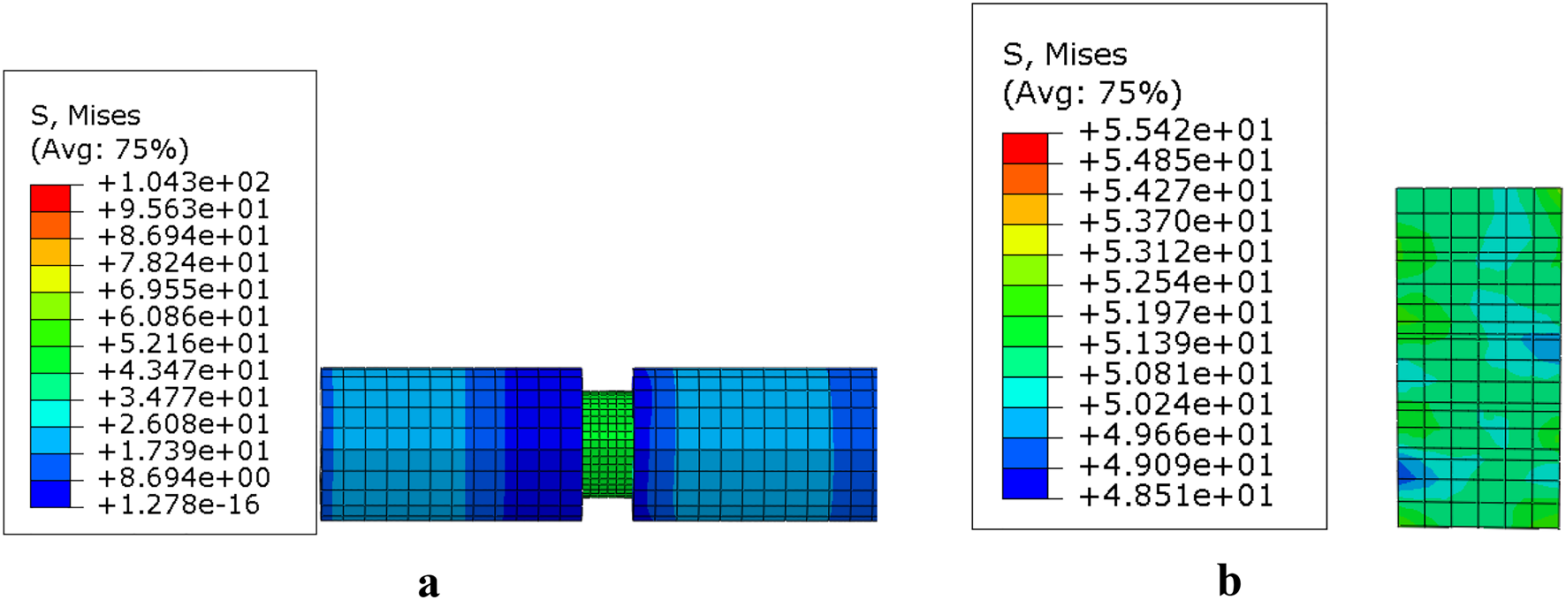
Von Mises stress cloud at different times: (**a**) *t* = 203 μs, (**b**) von Mises stress cloud on the specimen.

The strain rate-related behavior of the presented model is examined by uniaxial dynamic compression. At about 194 μs, the stress wave reaches the left end face of the specimen, which was the same as the transmitted time calculated using the elastic wave velocity ($c_{0} = \sqrt {{{E_{0} } \mathord{\left/ {\vphantom {{E_{0} } {\rho_{0} }}} \right. \kern-0pt} {\rho_{0} }}}$ = 5200 m/s, where $E_{0}$ = 73 GPa and $\rho_{0}$ = 2700 kg/m^3^ are Young’s modulus and mass density of the bar). Von Mises stress was nearly zero on the specimen, as shown in the Fig. 8a. The unit of von Mises stress cloud is MPa.

Part of incident wave is reflected back to the incident bar, and the remainder passes through the specimen to the transmission bar (the transmitted wave). To characterize the stress equilibrium, the relative stress difference was calculated as $\alpha = \Delta \sigma /\sigma_{T}$, where $\sigma_{T}$ is the mean Von Mises stress. The assumption of stress equilibrium was nearly satisfied if $\alpha \le 5\%$. The specimen achieves stress equilibrium at about 203 μs, as shown in Fig. 8b. Von Mises stress difference at each point does not exceed 0.7 MPa. The relative stress difference was < 5%. The simulation model determined the stress equilibrium.

**Figure 9:**
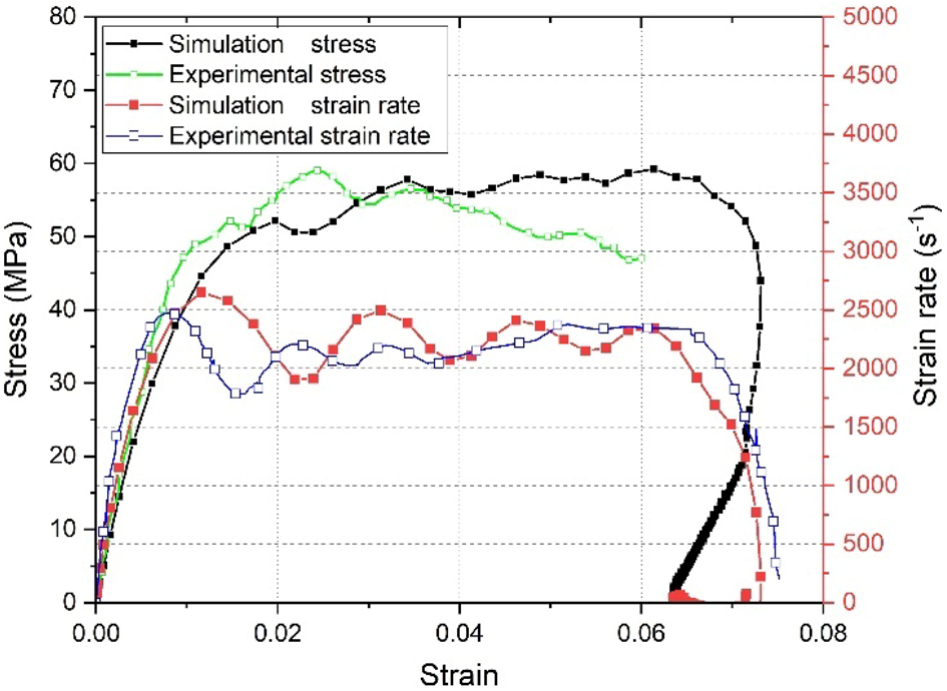
The comparison of stress–strain and strain rate curves changes during the simulation.

In fact, the stress–strain curve simulated by finite element model (FEM) is in good agreement with the experimental results under high strain rate. A point in the middle of the specimen surface was taken out, and the finite element simulation results of ABAQUS at strain rate of 2200/s were compared with the experimental results, as shown in Fig. 9.

**Figure 10:**
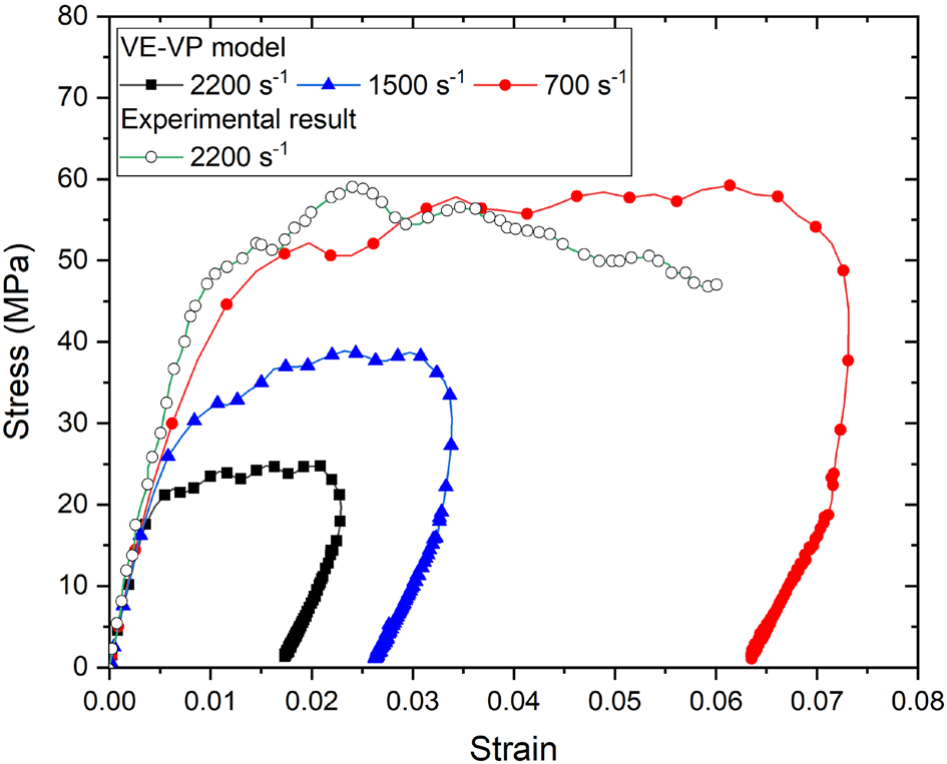
VE–VP model response to dynamic compression at different strain rates.

The uniaxial compressive stress–strain curves of PBX 9501 are compared by using the VE-VP model in Fig. 10. The strain rates in the loading direction were specified to be 700, 1500, and 2200/s for compression, respectively. The effect of rate on the material behavior are evident in the Figure. As shown in Fig. 10, the current model captures the main features of the experimental results, such as the initial slope, curvature and peak of the curve, quite well. As the plastic strain continued to increase, the plastic flow stress at different strain rates were parallel to each other, because the modulus of the hardening stage had the same value. Thus, this model could well describe the rate-dependent characteristics of PBX 9501.

### Analysis and discussion

The algorithm established in this paper can accurately and effectively simulate the dynamic mechanical properties of PBX9501. Compared with the methods reported in the existing literature^14,31–34^, there are two important differences: (I) For the discretization of the VE integral, an algorithm for the explicit update of a GMM was developed by using two schemes: constant VE strain rate, and mid-point rule. Due to small Δ*t* under impact loading, the methods are exact that the VE strain rate is constant over the step. Viscoplastic flow was considered in hyperbolic sinusoidal form. (II) The coupled algorithms of VE–VP model were developed by using the expression of incremental relaxation modulus. The methods are remarkably simple and form-identical to EVP provided that the constant elastic stiffness operator is replaced with $\tilde{L}_{ijkl}^{{}} \left( t \right)$ Eq. (31). (III) The display algorithm avoids the convergence in the calculation process, but this algorithm is satisfied by both integration methods for small Δ*t*. This algorithm is very suitable for predicting the mechanical properties of materials under impact loading. Ellsiepen et al.^35^ studied a class of diagonally implicit Runge–Kutta (DIRK) methods. This algorithm is much more advanced than the one presented in this paper. In a future work, it will be very interesting to develop a DIRK method instead, in order to achieve better accuracy and optimal time step control.

## Conclusions

The present work aimed at the coupled VE–VP modeling of the dynamic compressive behavior of PBXs. The total strain was assumed to be the sum of VE and VP components. The stress was related to the history of the VE strains by a GMM. A VP model was developed for the history of the VP strains. This study presented numerical and analytical results.

An algorithm for the explicit update of a GMM was developed by using two schemes: constant VE strain rate, and mid-point rule. Due to the time of loading was small, using the algorithm to predict the mechanical behavior was simple with high efficiency. It is worth to be recommended. If the Δt is not small, the algorithm can only be using under constant strain rate. The expression of incremental relaxation modulus was deduced by using the explicit algorithm proposed. The explicit algorithm of VE–VP model was developed by using the incremental relaxation modulus. The expression of VE–VP model was form-identical to classical EVP provided.

The VE–VP model was implemented in ABAQUS via a VUMAT. The VE, EVP, and VE–VP responses were also shown for comparison. The VE–VP model was more suitable for dynamic mechanical properties of PBX 9501. A SHPB numerical simulation was presented for the dynamic compressive responses of PBX 9501. The stress–strain curves of PBX 9501 were predicted by using the VE–VP model under different higher strain rates. Good convergence was achieved.

## Data Availability

All data generated or analyzed during this study are included in this published article.
