# Publisher Correction: A viscoelastic–viscoplastic constitutive model for polymer bonded explosives under low impact loading

**DOI:** 10.1038/s41598-023-30094-0

**Published:** 2023-03-01

**Authors:** Youcai Xiao, Zeyu Wang, Ruisheng Wang, Xiaowei Zhang, Chenyang Fan, Zhifang Wei, Yi Sun

**Affiliations:** 1 College of Mechatronic Engineering, North University of China, Taiyuan, 030051 China; 2 State Key Laboratory of Explosion Science and Technology, Beijing Institute of Technology, Beijing, 100081 China; 3 Science and Technology on Electromechanical Dynamic Control Laboratory, Xi’an, 710000 China; 4 Departments of Astronautic Science and Mechanics, Harbin Institute of Technology, Harbin, 150001 China

Correction to: *Scientific Reports* 10.1038/s41598-022-26525-z, published online 17 December 2022

The original version of this Article contained typographical errors.

Table 1 did not display correctly.

The original Table 1 and accompanying legend appear below.

**Table 1:** VE–VP algorithm.

**Input:** $\Delta e_{ij}^{ve}$, $\Delta \varepsilon_{V}^{ve}$, $\Delta t$; **Params:** $G^{\left( n \right)}$, $K^{\left( n \right)}$, $g^{\left( n \right)}$, $k^{\left( n \right)}$, $\sigma_{y}$, *h*, $\beta$, and *a* **Output:** ${{\varvec{\upvarepsilon}}}^{vp}$, ${{\varvec{\upvarepsilon}}}^{ve}$, $S_{ij}^{{}} \left( {t_{u + 1} } \right)$, $\sigma_{V}^{{}} \left( {t_{u + 1} } \right)$
1: Read the strain tensor at the (*u* + 1)*th* step 2: Compute the trial stress tensor using Eqs. (25) and (26) 3: Compute the incremental relaxation modulus: $$\left\{ \begin{array}{*{20}l} \tilde{G}^{{_{\left( n \right)} }} = \left[ {1 - \exp \left( { - \frac{\Delta t}{{\tau^{\left( n \right)} }}} \right)} \right]\frac{{g^{\left( n \right)} }}{\Delta t} \hfill \\ \tilde{K}^{\left( n \right)} = \left[ {1 - \exp \left( { - \frac{\Delta t}{{\tau^{\left( n \right)} }}} \right)} \right]\frac{{k^{\left( n \right)} }}{\Delta t} \hfill \\ \tilde{G} = G^{(\infty )} + \sum\limits_{n = 1}^{N} {G^{\left( n \right)} \tilde{G}^{{^{\left( n \right)} }} } \hfill \\ \tilde{K} = K^{(\infty )} + \sum\limits_{n = 1}^{N} {K^{\left( n \right)} \tilde{K}^{{^{\left( n \right)} }} } \hfill \\ \end{array} \right.$$ 3: Compute the von Mises yield function *f*: $f = \sigma_{eq}^{trial} - \sigma_{y} = \left( {\frac{3}{2}S_{ij}^{trial} :S_{ij}^{trial} } \right) - \sigma_{y}$ 4: If $f \ge 0$, compute the plastic strain rate; otherwise, go to step (1) 5: Use New iteration to compute the effective plastic strain increment as follows: $$\left\{ \begin{array}{*{20}l} \psi_{\alpha } = \sigma_{eq}^{trial} - 3\tilde{G}\Delta p - \sigma_{eq} \hfill \\ \psi_{\gamma } = \Delta p - \phi \Delta t \hfill \\ \phi_{\Delta p} = - 3\tilde{G}a\beta \cosh \left[ {\beta \left( {\sigma_{eq}^{trial} - 3\tilde{G}\Delta p - hp - \sigma_{y} } \right)} \right] \hfill \\ \phi_{\sigma } = - a\beta \cosh \left[ {\beta \left( {\sigma_{eq}^{trial} - 3\tilde{G}\Delta p - hp - \sigma_{y} } \right)} \right] \hfill \\ d\Delta p = \frac{{\phi_{eq} \psi_{\sigma } \Delta t - \psi_{\varepsilon } }}{{1 - \phi_{\Delta p} \Delta t + 3\tilde{G}\phi_{eq} \Delta t}} \hfill \\ \Delta p^{{\left( {r + 1} \right)}} = \Delta p^{(r)} + d\Delta p \hfill \\ \end{array} \right.$$ 6: Compute the nominal VP strain tensor as follows: $$\left\{ \begin{array}{*{20}l} {{\varvec{\upvarepsilon}}}^{vp} = \frac{3}{2}\Delta p\frac{{{\mathbf{S}}^{trial} }}{{\sigma_{eq}^{trial} }} \hfill \\ {{\varvec{\upvarepsilon}}}^{ve\_new} = {{\varvec{\upvarepsilon}}} - {{\varvec{\upvarepsilon}}}^{vp} \hfill \\ \end{array} \right.$$ 7: Compute the nominal stress tensor: $$ \begin{aligned} S_{{ij}}^{{new}} \left( {t_{{u + 1}} } \right) = & S_{{ij}}^{{\left( \infty \right)}} \left( {t_{u} } \right) + 2G^{{\left( \infty \right)}} \Delta e_{{ij}}^{{ve\_new}} \\ & + \sum\limits_{{n = 1}}^{N} {\exp \left( {\frac{{ - \Delta t}}{{\tau ^{{\left( n \right)}} }}} \right)S_{{ij}}^{{\left( n \right)}} \left( {t_{u} } \right)} \\ & + \sum\limits_{{n = 1}}^{N} {2g^{{\left( n \right)}} \frac{{g^{{\left( n \right)}} }}{{\Delta t}}\left[ {1 - \exp \left( {\frac{{ - \Delta t}}{{\tau ^{{\left( n \right)}} }}} \right)} \right]\Delta e_{{ij}}^{{ve\_new}} } \\ \end{aligned} $$ $$ \begin{aligned} \sigma _{V}^{{new}} \left( {t_{{u + 1}} } \right) = & \sigma _{V}^{{\left( \infty \right)}} \left( {t_{u} } \right) + 3K^{{\left( \infty \right)}} \Delta \varepsilon _{V}^{{ve\_new}} \\ & + \sum\limits_{{n = 1}}^{N} {\exp \left( {\frac{{ - \Delta t}}{{\tau ^{{\left( n \right)}} }}} \right)\sigma _{V}^{{\left( n \right)}} \left( {t_{u} } \right)} \\ & + \sum\limits_{{n = 1}}^{N} {3K^{{\left( n \right)}} \frac{{\tau ^{{\left( n \right)}} }}{{\Delta t}}\left[ {1 - \exp \left( {\frac{{ - \Delta t}}{{\tau ^{{\left( n \right)}} }}} \right)} \right]\Delta \varepsilon _{V}^{{ve\_new}} } \\ \end{aligned} $$

The original Article has been corrected.

